# Regeneration Filter: Enhancing Mosaic Algorithm for Near Salt & Pepper Noise Reduction

**DOI:** 10.3390/s25010210

**Published:** 2025-01-02

**Authors:** Ratko M. Ivković, Ivana M. Milošević, Zoran N. Milivojević

**Affiliations:** 1 Department of Software Engineering, Faculty of Economics and Engineering Management in Novi Sad, Cvecarska 2, 21000 Novi Sad, Serbia; 2 Department of Audio and Video Technologies, School of Electrical and Computer Engineering, Academy of Technical and Art Applied Studies, Vojvode Stepe 283, 11000 Belgrade, Serbia; ivana.milosevic@viser.edu.rs; 3 The Academy of Applied Technical and Preschool Studies, Aleksandra Medvedeva 20, 18000 Nis, Serbia; zoran.milivojevic@akademijanis.edu.rs

**Keywords:** digital image processing, noise reduction, regeneration filter, near salt & pepper noise, spatial density

## Abstract

This paper presents a Regeneration filter for reducing near Salt-and-Pepper (nS&P) noise in images, designed for selective noise removal while simultaneously preserving structural details. Unlike conventional methods, the proposed filter eliminates the need for median or other filters, focusing exclusively on restoring noise-affected pixels through localized contextual analysis in the immediate surroundings. Our approach employs an iterative processing method, where additional iterations do not degrade the image quality achieved after the first filtration, even with high noise densities up to 97% spatial distribution. To ensure the results are measurable and comparable with other methods, the filter’s performance was evaluated using standard image quality assessment metrics. Experimental evaluations across various image databases confirm that our filter consistently provides high-quality results. The code is implemented in the R programming language, and both data and code used for the experiments are available in a public repository, allowing for replication and verification of the findings.

## 1. Introduction

Although classified as a fundamental element of multimedia, the digital image, when viewed through the prism of digital signal processing, exhibits characteristics similar to those of other signal types. In every stage, be it generation, transmission, or processing, digital signals invariably incur a certain degree of noise. Thus, regardless of the conditions under which an image is created, it inherently contains some level of noise. This intrinsic noise presents a significant challenge within the field of Digital Image Processing (DIP), as it necessitates effective methods to mitigate or eliminate noise to an acceptable level. A prevalent type of noise encountered in digital images is Salt & Pepper (S&P) noise, which manifests as extreme pixel intensity values, specifically at 0 and 255 on the grayscale. The occurrence of S&P noise in images is spatially random, with affected pixels following a stochastic distribution, where the density of noise is quantified as a percentage. Initially, nonlinear filters were employed to address this type of noise; however, these filters indiscriminately processed all pixels within the image, including those unaffected by noise, which led to a reduction in image quality due to unnecessary processing of clean pixels.

Fundamentals of Nonlinear Digital Filtering is the first book to comprehensively explore and evaluate algorithms and applications in nonlinear digital filtering [1]. This seminal work serves as a reference handbook and textbook, catering to professors, researchers, engineers, and students alike. It establishes a foundational understanding of nonlinear filtering techniques and their practical implications, laying the groundwork for subsequent research endeavors. Impulse noise, particularly prevalent in image processing applications, presents a formidable obstacle to traditional filtering approaches. Paper [2] introduces innovative adaptive median filters designed to effectively remove impulses while preserving image sharpness. These algorithms demonstrate superior performance compared to conventional algorithms, offering promising solutions to the challenges posed by impulse noise corruption. Building upon the advancements in adaptive filtering, paper [3] proposes a novel decision-based algorithm for restoring images heavily corrupted by impulse noise. The algorithm exhibits remarkable efficacy in noise removal, surpassing the capabilities of standard median filters and adaptive schemes, while preserving image edges and details, even at high noise spatial density. Paper [4] presents an improved decision-based detail-preserving variational algorithm tailored for the removal of random-valued impulse noise. By integrating adaptive center weighted median filters and fast iteration strategies, the proposed algorithm outperforms existing algorithms in both qualitative and quantitative evaluations, offering expedited denoising solutions for practical applications. In parallel, the integration of infrared thermography instruments for PCB inspection underscores the interdisciplinary nature of noise reduction techniques. Paper [5] establishes a thermal model for PCB fault detection, leveraging advanced reconstruction algorithms to enhance temperature field quality. This approach showcases the versatility of noise mitigation strategies across diverse domains, highlighting their potential for real-world applications beyond traditional image processing contexts. Another research of impulse noise removal, paper [6] introduces a modified decision-based unsymmetrical trimmed median filter algorithm tailored for grayscale and color image restoration. This algorithm exhibits superior performance compared to conventional median filters, offering enhanced peak signal-to-noise ratios and image enhancement factors across various test scenarios. Paper [7] presents an effective noise removal algorithm utilizing switching median filtering techniques. By incorporating boundary discriminative noise detection, the proposed algorithm demonstrates superior noise suppression capabilities compared to traditional median filters, validating its efficacy through extensive simulation studies. Similarly, paper [8] introduces a novel switching median filter enhanced by boundary discriminative noise detection for denoising highly corrupted images. This algorithm achieves remarkable noise detection accuracy, outperforming existing algorithms while maintaining computational efficiency, thereby facilitating seamless integration into practical applications. Expanding the scope of noise removal beyond impulse noise, paper [9] proposes an efficient architecture for median filtering in image denoising applications. Leveraging decision filters and area-efficient designs, the proposed approach achieves significant improvements in denoising speed and peak signal-to-noise ratios, demonstrating its viability for real-world deployment. Paper [10] introduces a thresholding and regularization-based algorithms for salt and pepper noise removal, showcasing superior denoising performance compared to existing techniques. By leveraging total variation regularization and tailored noise detection strategies, the proposed algorithm offers robust solutions for noise reduction in large-scale image datasets. In parallel, paper [11] addresses limitations in switching median filters through innovative modifications to the boundary discriminative noise detection algorithm. Experimental evaluations highlight the effectiveness of these modifications in producing sharper images with enhanced noise suppression capabilities. Paper [12] presents a graph signal reconstruction-based approach for salt and pepper noise removal, offering a novel framework for image restoration. By modeling noise removal as a graph signal reconstruction problem, the proposed algorithm achieves superior denoising performance compared to state-of-the-art algorithms, demonstrating its potential for practical applications. Paper [13] introduces a two-step salt and pepper noise removal algorithm, combining median-type filtering with adaptive nonlocal bilateral filtering. By addressing errors inherent in median-type filtering, the proposed algorithm achieves superior noise reduction while preserving image details, offering a compelling solution for challenging noise removal scenarios. Although all the aforementioned solutions for reducing S&P noise have their advantages and contribute significantly to the field of digital image processing, they are not yet ideal solutions. Nearly all proposed models fail to produce satisfactory results beyond 30% noise. This manuscript provides insight into a novel filter solution for S&P noise reduction based on spatial domain processing, with a particular emphasis on statistical processing of numerical data (pixel values), which can be reliably utilized even in reducing images with 70% noise spatial density. Additionally, it is noteworthy that by reusing images, there is no loss in quality, thus enabling the reconstruction of images even with a 90% noise spatial density.

The proposed algorithm, named the Regeneration Filter, utilizes multi-directional iterative image processing to identify and replace noise-affected pixels while preserving the structural details of the image. The core idea of the algorithm is based on analyzing the local context within 3 × 3 matrices, where noise-affected pixels are replaced with values from healthy neighboring pixels. To define specific conditions, the proposed algorithm employs a hybrid edge detection approach within the mosaic methods framework [14]. The algorithm processes the image in multiple directions (horizontal, vertical, diagonal, and reverse order), enabling the detection and regeneration of noise-affected pixels that were not identified in previous passes. This iterative approach ensures effective noise reduction even at high noise densities, with minimal degradation of image quality. The algorithm was implemented using the R programming language, leveraging its capabilities for data manipulation and matrix processing, which facilitates easy reproducibility and further analysis of the results. Near Salt & Pepper (nS&P) noise is a specific type of digital image noise characterized by near extreme pixel values, typically at the grayscale levels of 0 (black) and 255 (white). This noise appears as sparse white and black pixels distributed randomly throughout the image. The presence of nS&P noise can significantly degrade image quality, making it challenging to accurately process and analyze the affected images. Traditional filters often struggle with effectively removing this noise without affecting the uncorrupted pixels. Advanced algorithms, such as the Regeneration Filter, are designed to selectively target and reconstruct the noisy pixels, ensuring the integrity of the image is preserved even at high noise levels. This manuscript addresses the challenge of reducing nS&P noise, particularly focusing on the noise that occurs in the vicinity of these near extreme values, as well as the extreme values themselves. To ensure the manuscript maintains a clear and logical flow, the structure has been organized so that the proposed algorithm is first explained in detail, followed by comparisons with other methods in the experimental results section. This approach allows the introduction to focus on establishing the context and objectives of the proposed method, while detailed evaluations and comparisons are presented alongside the experimental analysis. This structure was chosen to enhance readability and provide a cohesive discussion of the results in one place.

This paper is organized as follows: Section 2 provides a detailed description of the methodology behind the implementation of the Regeneration filter, including the algorithm for detecting and restoring pixels affected by near Salt & Pepper (nS&P) noise. Section 3 addresses specific processing conditions, while Section 4 presents the mathematical formalization of the filter, including the algorithmic approach for image restoration. Finally, Section 5 reports the simulation results and discussions, with a comparison against existing noise reduction methods. In Section 6, we conclude the paper and outline potential future research directions.

## 2. Implementation Methodology

The Regeneration filter represents a novel approach to the removal of nS&P noise from digital images. Aptly named after its property of “regenerating” the image, this filter selectively processes only the pixels affected by extreme values, characteristic of this type of noise, reconstructing them based on the “healthy” surrounding pixels. This eliminates the potential for damage to the parts of the image that are not affected by noise, providing an effective solution for images with high levels of spatial densities, without compromising quality.

Samples for analysis by the proposed filter were based on the images:

- Different resolutions (using processing of submatrices with repetition gives additional precision of the filter. Regardless of the square submatrix, the quality of processing does not depend on the resolution. Test resolutions covered the range from 262,144 P to 48 MP);
- Different bit depths (a test of this type provided confirmation that the filter gives very good results with both 24-bit and 32-bit recording);
- Different formats, to determine the quality, regardless of the type of compression;
- Generated for computer animation—to determine the quality of images created in special conditions;
- Generated CCD and CMOS sensors—real images from cameras/mobile phones to show the quality of filters in various real situations in which images are taken and
- Characteristic images in the field of digital image processing so that the results are measurable with other filters designed for this type of noise.

**Figure 1:**
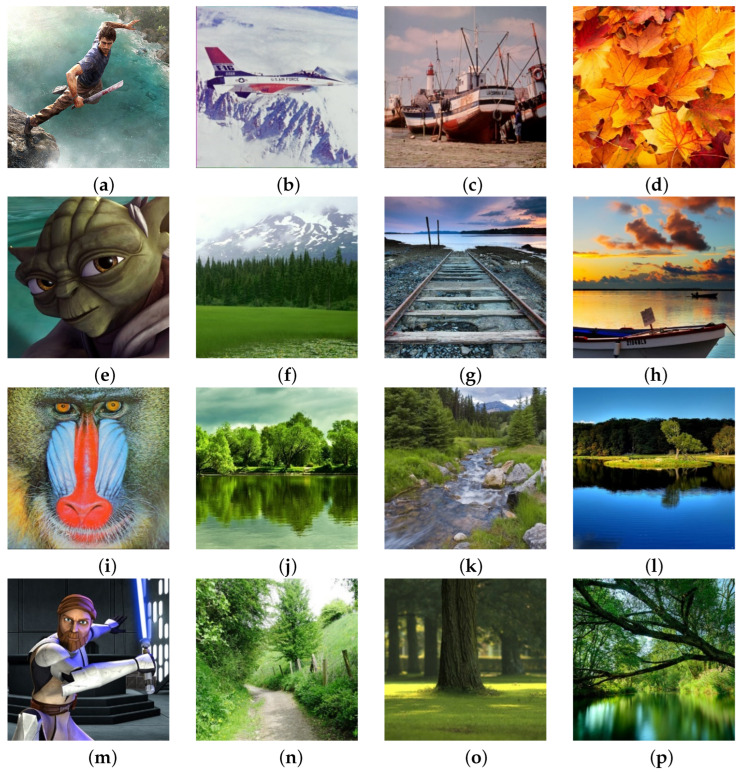
Original samples of digital images.

**Figure 2:**
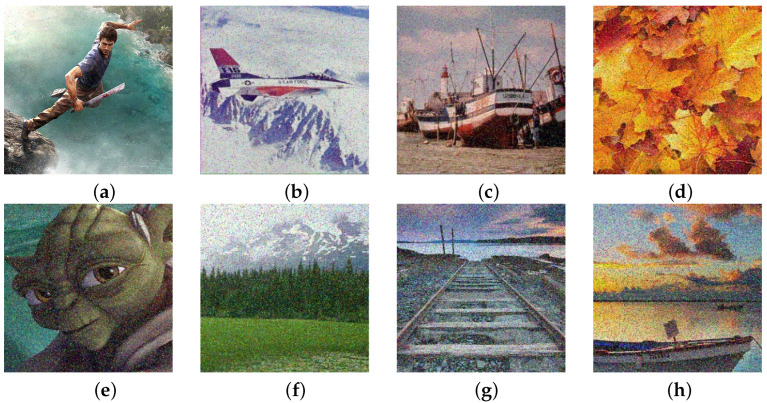

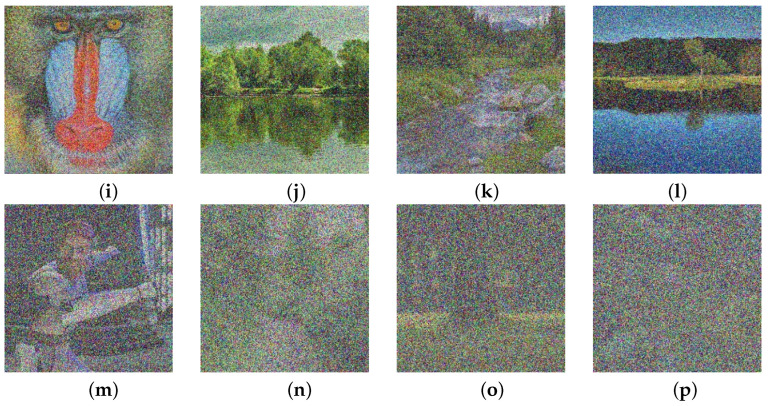
Digital image samples with added nS&P noise: (**a**) 1%, (**b**) 2%, (**c**) 3%, (**d**) 4%, (**e**) 5%, (**f**) 7.5%, (**g**) 10%, (**h**) 10%, (**i**) 30%, (**j**) 40%, (**k**) 50%, (**l**) 50%, (**m**) 70%, (**n**) 80%, (**o**) 80%, (**p**) 90%.

Figure 1 shows the part of the sample that was subjected to the processing process, while the entire database of test images was taken from the address: https://www.imageprocessingplace.com/root_files_V3/image_databases.htm (accessed on 14 December 2024). Regeneration filter as one of the results of the mosaic algorithm, presents it in full light and shows all the advantages of the proposed algorithm. Precisely with its ability to adapt to the needs of digital image processing, the mosaic algorithm provides very good results in almost all areas, as well as in this situation when reducing nS&P noise. It’s important to note that all images in this manuscript are standard images commonly utilized in works related to digital image processing, ensuring the measurability of this filter’s results against similar studies in this field. Samples of original images of various resolutions were subjected to the addition of nS&P noise using the established function within the R programming language. The original image underwent noise corruption by implementing the command for adding 1% nS&P noise, transitioning from Figure 1 to Figure 2. Subsequently, the process was iterated across the sample with incremental additions of nS&P noise spatial densities: 2%, 3%, 4%, 5%, 7.5%, 10%, 30%, 40%, 50%, 70%, 80%, and 90%. Figure 2 displays the resulting samples with noise additions ranging from 1% to 90%. Additionally, each sample with added noise serves to demonstrate the effects of varying spatial density of noise on image quality and the performance of the Regeneration filter.

Regeneration filter is applied over all images with added noise. The input to the filter is an image that contains noise, and the result of processing by the filter is an image of the same format and resolution as the input over which it was applied in the Regeneration filter. All images utilized in the analysis were stored in TIFF format for the purpose of mitigating the influence of compression on visual and numerical outcomes. The output image was compared with the original image without noise in order to determine the quality of the proposed filter.

## 3. Defining Special Processing Conditions

A new model of partial filtering in the implementation of algorithms for edge detection and digital image segmentation - mosaic algorithm, allows the image, using a hybrid model of edge detection (using a modified Weighted filter, image negative and providing the user to define the detection threshold) segments (parts of a mosaic) whereby the desired processing can be applied over each segment of the image with different or the same parameters.

**Figure 3:**
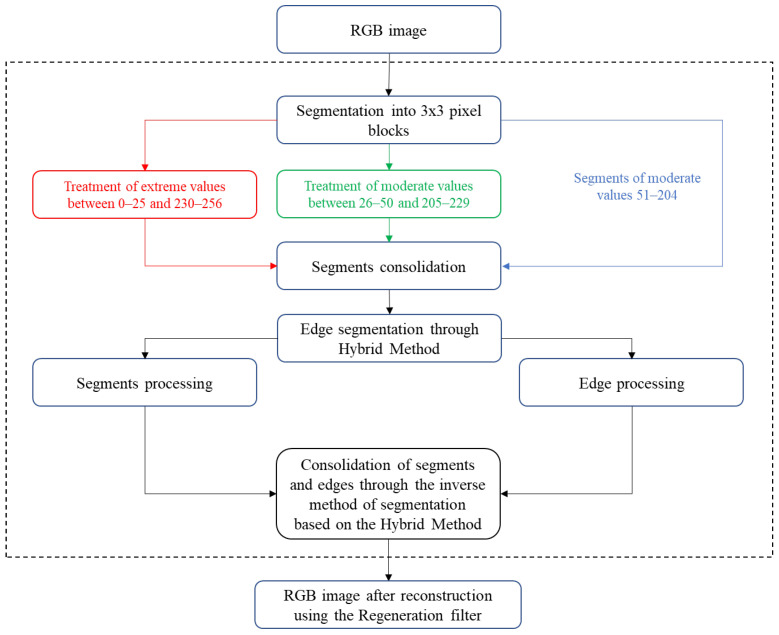
Mosaic algorithm optimized for nS&P noise.

Just as defined in the block diagram of the mosaic algorithm (Figure 3), the processing segments in this case will be submatrices 3 × 3. As explained in papers dealing with filter design [14,15]. When it comes to this type of problem, it is best to work with square submatrices. Vector analysis, where the pixels of a digital image are processed row by row, practically eliminates the connection of the surrounding columns with those that are processed, which can cause rough transitions in the images, and leads to an error in edge detection. The 3 × 3 processing matrix is the optimum on which more than 90% of the noise reduction filter is based [16]. If, for example, 2 × 2 submatrices are used and it is assumed that the reconstruction is done only on the basis of one “correct” pixel, it is possible to reconstruct the image up to a maximum of 75% of the damage. On the other hand, if it is a 3 × 3 matrix processing, and an attempt is made to reconstruct an image with a minimum of one “correct” pixel, we are talking about a potential reconstruction of approximately 90%. Another advantage of this model is the center pixel, with a position of five in that array of nine pixels. The median and all derivatives of this filter base their work on processing in relation to the value of the central pixel. Other 4 × 4 or 5 × 5 matrices give a higher degree of error, so they are not used often. Thus, for a 4 × 4 processing matrix, 16 pixels are processed at the same time, or for a 5 × 5 matrix, 25 pixels are processed at the same time. Such processing algorithms are used at extremely high noise spatial densities, while at low spatial densities they give a higher degree of error.

Another very important item in this part is the overlap of submatrices in processing. If a 3 × 3 matrix is used, the process involves processing 9 pixels. Processing without overlapping 18 pixels would be processed through two independent 3 × 3 iterations, while in the case of overlapping 18 pixels it would be processed through four independent iterations. The number of independent operations in one image processing has been doubled. This fact significantly increases the complexity of this algorithm, but on the other hand increases the efficiency of the filter. Thus, for example, if the noise spatial density in the figure is 90%, it can also be assumed that in the observed submatrix 3 × 3 at least one pixel is not covered by noise, so that based on one pixel, a “new” environment of that pixel can be reconstructed. This extreme example with a noise spatial density of 90% is the best explanation why the 3 × 3 pixel submatrix was chosen. But if by chance it happens that all the pixels of the first submatrix are detected as noise, already in the next step 6 pixels of the first submatrix will be found in the processing, which increases the efficiency of the proposed filter. The next step in the processing is the synchronization of the intermediate results of the submatrix processing. It often happens that there are extremely large differences between the pixel values after processing the submatrix. Therefore, spline interpolation is used to regulate the transition, due to the low degree of error, because lower degrees of polynomials are applied. “Fine transitions” between pixels should be used to replace pixels that are “damaged” by noise. Only in the next step over such an image is a hybrid model of edge detection used in order to further emphasize the edges. This procedure is performed by summing the detection results with the image. After the mentioned procedure, through a series of parameters for assessing the quality of the digital image, the obtained results are described.

The algorithm traverses all pixels of the image and treats only those pixels with extreme values using the algorithm, as indicated in Figure 3, performing this process in four directions to remove noise from all parts of the image. This selective processing of pixels with extreme values represents a key optimization of the proposed Regeneration filter compared to traditional approaches that process all pixels indiscriminately. First, the algorithm iterates through all pixels in the horizontal direction (rows), checking whether the value of each pixel exceeds a threshold of 254 or falls below a threshold of 2, indicating the presence of nS&P noise. If a pixel is affected by noise, its value is replaced with the value of a “healthy” pixel in the same row. This process is repeated for each row, except for the first row which lacks preceding pixels for reference. Next, the same procedure is applied in the vertical direction (columns), where noise-affected pixels are replaced with values of “healthy” pixels, except for the first column. Subsequently, the algorithm moves on to the third processing direction, replacing noise-affected pixels with values of pixels located to their right in the same row. The fourth and final direction involves replacing noise-affected pixels with values of pixels below them in the same column. Such a four-directional approach ensures that each noise-affected pixel is treated and replaced with an appropriate value based on surrounding “healthy” pixels, achieving effective noise removal from all parts of the image. The key advantage over traditional filters is that the processing focuses solely on noise-affected pixels untouched.

The algorithm employs an iterative image processing approach across multiple directions (horizontal, vertical, diagonal, and reverse orders). This multi-directional processing enables the identification and replacement of noise-affected pixels that may not have been detected in previous passes due to the specific noise distribution or the context of pixels within the 3 × 3 matrix.

**Multiple Passes for High Noise Levels**

The proposed algorithm demonstrates a step-by-step processing logic. In each pass, noise-affected pixels are replaced with values from “healthy” neighboring pixels. However, in cases of high noise density, it is possible for the entire 3 × 3 submatrix to be affected by noise during the initial pass. In such cases, the first pass may fail to replace all noise-affected pixels because none of the neighboring pixels are sufficiently “healthy” to provide accurate replacements. Therefore, the algorithm relies on subsequent passes in different directions, utilizing pixels that were already regenerated in previous iterations. This iterative process gradually reduces noise levels over multiple passes.

**Adaptation to Local Context**

The proposed algorithm implements a logic where extreme pixel values (≤2 or ≥254) are replaced with values from “healthy” neighbors, depending on the processing direction. Multi-directional processing (e.g., top-down, left-right, diagonal) allows for the generation of improved replacements. This is particularly critical in scenarios where noise is randomly distributed, as certain iterations may have better chances of identifying "healthy" pixels in alternate directions. Additionally, restoring values in one direction improves the quality of the neighboring context for subsequent passes in other directions.

**Dependence on Noise Levels**

The algorithm is designed to handle high noise levels (up to 97%). In cases where the spatial distribution of noise is dense, it is possible for none of the pixels in a submatrix to be “healthy.” In such scenarios, the algorithm requires multiple iterations, leveraging neighboring submatrices regenerated in prior passes to reconstruct the image.

*Example*: If a horizontal pass fails to replace a noise-affected pixel because all its horizontal neighbors are also affected by noise, a diagonal pass can utilize newly regenerated values from the previous iteration to achieve successful replacement.

**Regeneration Efficiency**

The algorithm ensures that each iteration improves the local context of the image, and repeated passes in various directions do not degrade the quality of previously regenerated pixels. Instead, it builds upon them to further reduce noise and improve the overall image quality.

The algorithm detects new noise-affected pixels in each processing step, including the third and fourth steps, as each pass utilizes a different direction for analysis and replacement. In the initial steps, noise-affected pixels are replaced based on the available “healthy” pixels in a specific direction (horizontal or vertical). However, due to the specific distribution of noise or a high noise density, some pixels may remain undetected or insufficiently regenerated in the first passes. For example, if the neighboring pixels in the horizontal direction are also affected by noise, the algorithm cannot generate an accurate replacement. Nevertheless, in subsequent passes (diagonal or vertical directions), newly regenerated “healthy” pixels may emerge, enabling successful detection and replacement. Consequently, the third and fourth steps do not repeat previous replacements but rather identify additional noise-affected pixels that were not detected in the initial pass. This iterative process is essential for ensuring complete image reconstruction under high noise conditions, as each pass contributes to improving the local context of the image and allows the algorithm to gradually regenerate the affected regions.

The algorithm does not utilize preceding pixels as a reference in the first row or column because these pixels lack neighboring pixels in the designated processing direction (horizontal or vertical) that could serve as replacements. In such cases, the algorithm relies on the next available pixels in the second row or column to replace the noise-affected pixels. This approach is particularly critical in high-noise scenarios, where all pixels in the first row or column might be affected by noise, rendering reliable regeneration during the initial pass impossible. However, if “healthy” pixels exist within the same row, they are employed in subsequent iterations and processing directions, where diagonal or reverse directions facilitate the detection and replacement of noise-affected pixels based on neighboring submatrices. This method ensures that the algorithm can reliably process the image edges without introducing additional artifacts, while the iterative passes allow for the use of regenerated pixels to further replace noise in subsequent steps.

## 4. Mathematical Framework

Near Salt & Pepper (nS&P) noise is a specific type of noise that affects not only the extreme pixel values (0 or 255), but also those in their immediate vicinity, i.e., pixels whose values are near these extremes. Formally, this noise can be described as a set of pixels with values within the intervals [0, $T_{1}$] and [$T_{2},255$], where $T_{1}$ and $T_{2}$ are predefined thresholds that define the range of values close to the minimum and maximum intensities.

### 4.1. Formalizing Noise

Let I(x,y) be the pixel intensity at position (x,y) in the image *I*. Pixels affected by nS&P noise can be formally defined as

(1) $$I_{noisy}\left(x,y\right)=\left\{\begin{array}{cc} I(x,y), & \mathrm{with}\;\mathrm{probability}\;1-p\\ u\in [0,T_{1}], & \mathrm{with}\;\mathrm{probability}\;p/2\\ v\in [T_{2},255], & \mathrm{with}\;\mathrm{probability}\;p/2 \end{array},\right.$$

where $T_{1}$ and $T_{2}$ are thresholds near the minimum and maximum pixel values, and *p* represents the noise density. In this model, pixels *u* take values near 0, while pixels *v* take values near 255. This allows the nS&P noise model to include pixels close to the extreme values, not just those exactly at 0 and 255.

### 4.2. Noise Detection

The detection of pixels affected by nS&P noise is carried out using a binary mask M(x,y) which marks pixels that fall within the noise region. Formally, the mask is defined as:

(2) $$M\left(x,y\right)=\left\{\begin{array}{cc} 1, & \mathrm{if}\;I\left(x,y\right)\in \left[0,T_{1}\right]\cup \left[T_{2},255\right]\\ 0, & otherwise \end{array}.\right.$$

This mask differentiates “noisy” pixels from “healthy” pixels in the image. Pixels with M(x,y)=1 are treated as affected by noise, while those with M(x,y)=0 remain unchanged.

### 4.3. Pixel Restoration Algorithm

Pixels marked as noisy are restored based on their neighboring “healthy” pixels. The regeneration algorithm uses a local window N(x,y)—the pixel’s neighborhood—to calculate the restored pixel value. The neighborhood N(x,y) is defined as a matrix of size *k* × *k*, where *k* is the window size (e.g., *k* = 3 for a 3 × 3 window):

(3) N(x,y)={I(i,j),i∈[x−k,x+k],I(i,j),j∈[y−k,y+k]}.

For each pixel I(x,y) marked by M(x,y)=1, its intensity is restored based on the pixels in its neighborhood N(x,y) that are not affected by noise. We define the subset $N^{\prime }\left(x,y\right)$ of neighboring pixels that are not affected by noise as

(4) $$N^{\prime }\left(x,y\right)=\left\{I\left(i,j\right),I\left(x,jy\right)\in \left[0.T_{1}\right]\cup \left[T_{2},255\right],\left(i,j\right)\in N\left(x,y\right)\right\}.$$

Unlike traditional filters, the Regeneration filter does not use median filtering. Instead, the restoration of each noisy pixel is based on a specific algorithmic strategy that analyzes the available pixel values in the neighborhood. For each noisy pixel I(x,y) the algorithm examines the neighboring pixels within N(x,y). These neighboring pixels are evaluated to determine whether they are free from noise (i.e., if M(i,j)=0. In the Regeneration filter, the restoration of noisy pixels involves averaging the values of neighboring, non-noisy pixels using a set of weights $w_{i,j}$, where (i,j) represents the coordinates of the neighboring pixels. These weights determine how much influence each neighboring pixel has on the restoration of the noisy pixel, often assigning higher weights to closer or more relevant pixels.

The restoration of a noisy pixel I(i,j) based on its non-noisy neighbors is done by calculating a weighted average. This process consists of two main steps:

1. Sum the contributions of all neighboring pixels that are not affected by noise:
  (5) $$S\left(x,y\right)=\sum_{(i,j)\in N(x,y),M(i,j)=0}\omega_{i,j}\cdot I\left(i,j\right),$$
  where S(x,y) represents the sum of the weighted pixel values in the neighborhood N(x,y). Here, $\omega_{x,y}$ is the weight assigned to pixel I(i,j) and M(i,j)=0 ensures that only non-noisy pixels are included in the sum.
2. Normalize the sum by dividing it by the sum of the weights:
  (6) $$I_{new}\left(x,y\right)=\frac{S(x,y)}{\sum_{(i,j)\in N(x,y),M(i,j)=0}\omega_{i,j}},$$
  where the denominator ensures that the sum is properly scaled according to the total weight of the valid neighboring pixels. This step results in the final restored value $I_{new}\left(x,y\right)$.

This two-step process allows the algorithm to take into account the varying importance of each neighboring pixel during the restoration of the noisy pixel. The weights $\omega_{i,j}$ can be uniform (i.e., $\omega_{i,j}=1$ for all neighboring pixels) or they can vary depending on the distance of each neighboring pixel (i,j) from the pixel being restored (x,y). For example, a common choice for non-uniform weights is the Gaussian weight function, which assigns higher weights to pixels closer to (x,y). The Gaussian weight function is given by:

(7) $$\omega_{i,j}=e^{-\frac{d^{2}\left(i,j\right)}{2\sigma^{2}}},$$

where d(i,j) represents the Euclidean distance between pixel (i,j) and (x,y), and σ is the standard deviation that controls the spread of the weights.

The restoration of noisy pixels in the Regeneration filter involves a two-step process: first, summing the weighted contributions of valid neighboring pixels, and second, normalizing the sum by the total weight of those neighbors. If no valid neighbors are available in the immediate vicinity, the algorithm expands the neighborhood and uses interpolation to restore the pixel values. This approach ensures that the restored pixel values are both accurate and contextually sensitive to the surrounding image structure.

In the experimental evaluation, additional analysis of the key algorithm parameters, such as filter size, thresholds $T_{1}$ and $T_{2}$ (Equation (1)), and the weighting parameter w (Equation (5)), would provide a more detailed understanding of their contributions to the algorithm’s efficiency. The filter size, represented by matrices of dimensions 3 × 3, 5 × 5, 7 × 7, or 9 × 9, plays a crucial role in the pixel regeneration process. The 3 × 3 submatrix has been selected as the optimal solution because it offers the lowest error in detecting and replacing noise-affected pixels—it provides sufficient local context for accurate regeneration while minimizing the risk of introducing additional errors, which can occur when processing larger matrices. For instance, while 5 × 5 or 7 × 7 matrices provide broader coverage, they increase the algorithm’s complexity and the risk of incorrect replacements, especially at higher noise levels. In contrast, the 3 × 3 matrix ensures high reliability with minimal processing errors.

Thresholds $T_{1}$ and $T_{2}$ play a significant role in defining the range of values the algorithm identifies as noise. Lower threshold values reduce the risk of replacing correct pixels, while higher thresholds allow the algorithm to detect noise even in images with extreme pixel values. Precise calibration of these thresholds could further enhance the algorithm’s accuracy across different scenarios.

The weighting parameter w, which determines the significance of neighboring pixels in the regeneration process, is critical for balancing the contributions of closer and more distant neighbors. Higher w values amplify the influence of closer pixels, while lower w values enable a more even contribution from the entire neighborhood, which is particularly advantageous for images with randomly distributed noise.

## 5. Results and Discussion

### 5.1. Results

The analysis of the results was carried out through the analysis of each parameter separately, where the filter will be evaluated according to the quality in relation to the processed images. On the other hand, each parameter was treated through a standard deviation of images with the same degree of damage. The StD parameter will assess the stability of the filter for various spatial densities.

**Figure 4:**
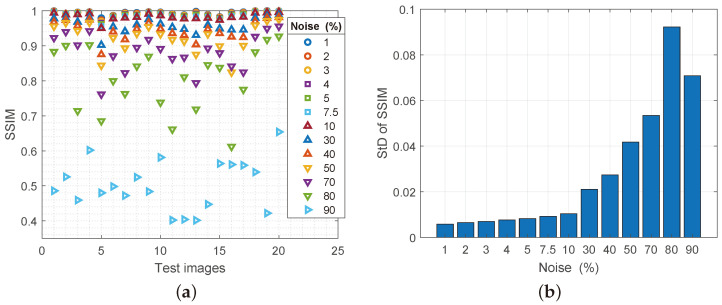
(**a**) Values of the SSIM parameter for reconstructed images with increasing noise spatial density, (**b**) values of the standard deviation of the SSIM parameter.

The assessment of the structural similarity (SSIM) of the original and the reconstructed image is given in Figure 4a. Samples are presented horizontally, and the degree of similarity according to SSIM is presented vertically. Different colors on the chart indicate different noise spatial densities, from 1% (0.01 on the chart) to 90% (0.9 on the chart). As can be seen from the graph, the degree of similarity of the reconstructed images to the noise percentage of 70% (0.7 on the x-axis of the graph) is over 80% on the y-axis of the graph of similarity according to SSIM. For a noise spatial density of up to 50%, the percentage of reconstruction and similarity with the original image is over 90%. This is the first proof of the high quality of the proposed filter. Figure 4b gives the average value of the standard deviation per certain noise level (*x*-axis) in relation to the average degree of similarity according to SSIM (*y*-axis). An increase in the degree of noise causes an increase in the standard deviation for the processed images. However, up to a degree of noise spatial density of up to 10%, the level of standard deviation is practically unchanged. In these situations, the proposed filter gives the best results in all observed conditions, so the average deviation is up to 10% noise, 1% difference compared to the original images. From 30% damage to 70% damage, this deviation is up to 5.2% compared to the original images. This relationship between the degree of noise and the similarity reconstructed with the original image unequivocally indicates a high degree of stability of the proposed filter in all conditions. When it comes to 80% damage, the degree of change of the standard deviation is 9.1% (on the y-axis between 0.08 and 0.1) and here is the largest deviation of the proposed filter. This noise spatial density when processing a 9-pixel submatrix contains a maximum of 1–3 pixels that are not affected by nS&P noise.

Theoretically, in an ideal distribution with a noise spatial density of 90%, each submatrix would ideally contain at least one pixel devoid of noise, thus enabling reconstruction based on it. However, in practice, this situation will almost never happen, so the processing is based on overlapping matrices. During this analysis, the noise density was of such spatial density that at 80% damage, the range of submatrices containing at least one noise-free pixel varied from 54 to 67% in the samples. The degree of damage of 90% provides reconstruction according to SSIM from 40 to 65%, so the degree of standard deviation for this degree of noise is 5.03%. The degree of noise is so high that the analysis must contain another repetition of the whole process, only then the standard deviation has values of 13.07%.

**Figure 5:**
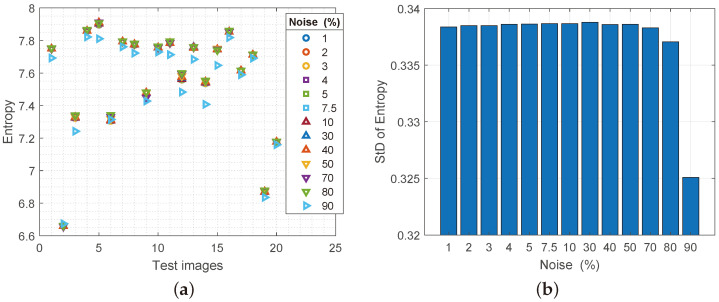
(**a**) Values of the entropy parameter for reconstructed images with increasing noise spatial density, (**b**) values of the standard deviation of the entropy.

All original images have an entropy of 8 or approximately 8 bits before any processing. Figure 5a shows the entropy values after image processing with added S&P noise. A higher degree of entropy corresponds to a higher “potential” of the image for later processing. All images that were subjected to analysis have a degree of entropy over 6.6 bits, which in theory is considered a very high value. Another confirmation of the stability of the attached filter can be found in Figure 5b. The changes in entropy levels for the complete analysis to which the Regeneration filter was subjected are very low, practically negligible up to 80% of the damage to the image by nS&P noise.

**Figure 6:**
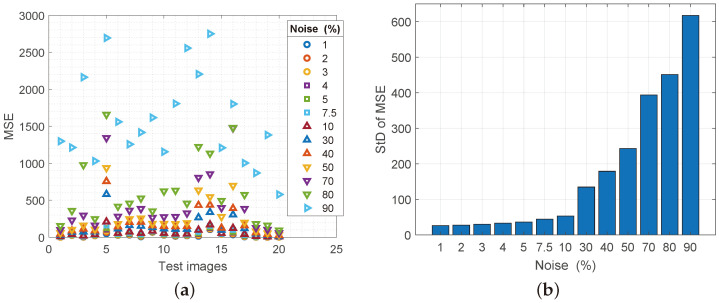
(**a**) Values of the MSE parameter for reconstructed images with increasing noise spatial density, (**b**) values of the standard deviation of the MSE parameter.

The mean square error (MSE) error provides the ratio of how much noise the image generated after processing. Since the noise-reducing filter is analyzed, values that weigh zero are acceptable on the scale. Figure 6a shows that for the largest percentage of processed images, MSE values are below 1000 dB, which is almost negligible information for high-resolution images, which were the object of processing. Below 500 dB MSE is almost 87% of the processed images, which confirms the claim of measurement by the algorithm of structural similarity. For StD measurement for MSE the same conclusion can be taken as for StD for SSIM, as seen in Figure 6b. The deviation to the noise level of 30% is almost negligible. StD values for MSE from 40 to 90% of the noise spatial density are below the statistical error level for this parameter. Also, as in previous analysis situations for StD, the Regeneration filter shows exceptional stability in operation.

**Figure 7:**
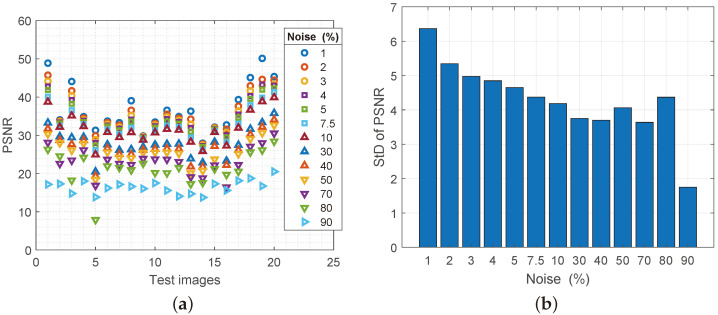
(**a**) Values of the PSNR parameter for reconstructed images with increasing noise spatial density, (**b**) values of the standard deviation of the MSE parameter.

The peak signal-to-noise ratio (PSNR) is not an absolute relevant parameter for quality assessment, but when it comes to describing a new filter, it gives a complete picture. In the theoretical considerations that can be read in [17,18], the desirable ratio between the original and the processed image is between 20–50 dB. As can be seen from the attached Figure 7a, approximately 87% of the processed images meet the theoretical requirements of high quality. Taking into account that there is no initial value for PSNR, the results of the change of StD for PSNR do not exceed 6.2, which can be seen in Figure 7b. On the other hand, very low StD oscillations ranging from 1.8 to 6.2 also favor the stability of the Regeneration filter.

**Figure 8:**
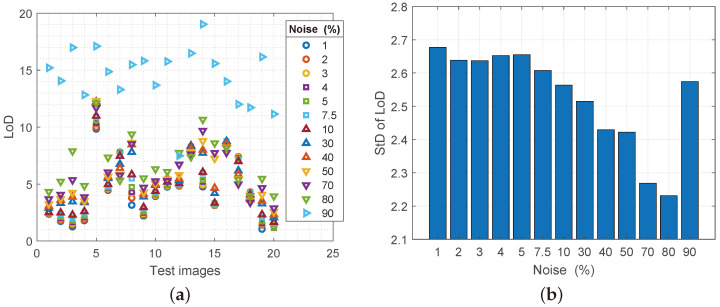
(**a**) Values of the LoD parameter for reconstructed images with increasing noise spatial density, (**b**) values of the standard deviation of the LoD parameter.

The level of details according to DCT (LoD DCT), as shown in Figure 8a, shows that as the noise in the image increases, so does the level of detail in it. Although increasing the level of details is treated as a positive effect (the image is clearer, the edges are clearer, etc.), here it cannot be taken as a positive side, because the increase in noise spatial density in the image causes an increase in details. That is why almost all image samples at a noise spatial density of 30% and more fall into the category of images with an extremely high level of details. StD changes for DCT are given in Figure 8b. The graph shows that all analyzed samples from extremely high levels of values were reduced to medium or low level of details, which coincides with the original images.

**Figure 9:**
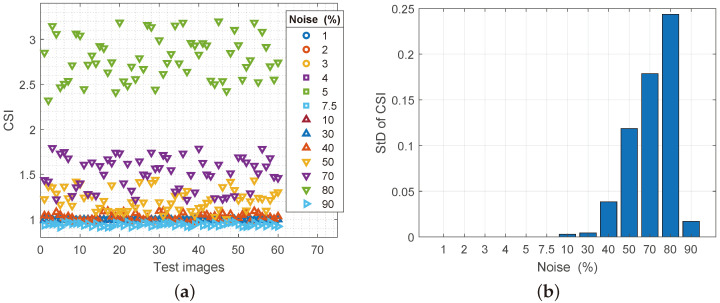
(**a**) Values of the CSI parameter for reconstructed images with increasing noise spatial density, (**b**) values of the standard deviation of the CSI parameter.

The comparison of CSI parameters is a comparison of the similarity of each component separately, the original sample with its pair in the reconstructed image [14]. Thus, the R component of a sample of an image will be compared with the R component of the reconstructed image, respectively for the G and B channels. That is why Figure 9a shows 120 comparisons grouped according to the degree of noise. The ideal situation is when the original and the reconstructed image have a value of 1 because it indicates the complete similarity of these two images in the structure of the component. Values above 1, in this case, indicate a certain degree of noise that is in the image after reconstruction. The results of CSI factor measurements indicate that a slight increase above 1 can be observed in the reconstructed images above 40% of the added noise. This leads to the conclusion that the success of Regeneration filters up to 40% of the added noise is almost 100%. The changes in the CSI parameter (Std of CSI) shown in Figure 9b once again confirm the stability in operation under all conditions up to a noise spatial density of 40%. As in the previous analysis with the SSIM parameter, so with the standard deviation for the CSI parameter, the level of standard deviation increases with noise growth. A similar conclusion can be drawn as for StD for SSIM at a noise spatial density of 90%. The noise level is so high that another iteration of the Regeneration filter is necessary to further remove some of the noise. After another iteration, the value of the standard deviation is 0.

**Figure 10:**
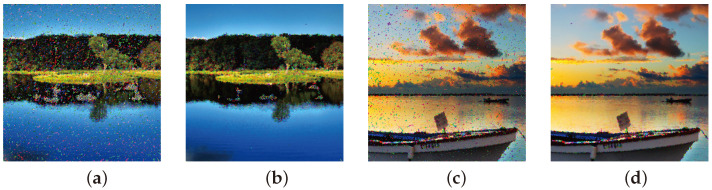
Images with added nS&P noise and result of reduction by regeneration filter for 90% noise spatial density for: (**a**,**c**) with first treatment and (**b**,**d**) with second interaction.

Specifically, Figure 10a,c depict the outcome of the initial treatment by the Regeneration filter, following the addition of 90% noise spatial densities to images from Figure 1l,h. Conversely, Figure 10b,d show case the results after the second interaction with the Regeneration filter. It is evident from Figure 10b,d that there is no loss of image quality after the repeated use of the filter, and the results indicate further noise reduction.

**Figure 11:**
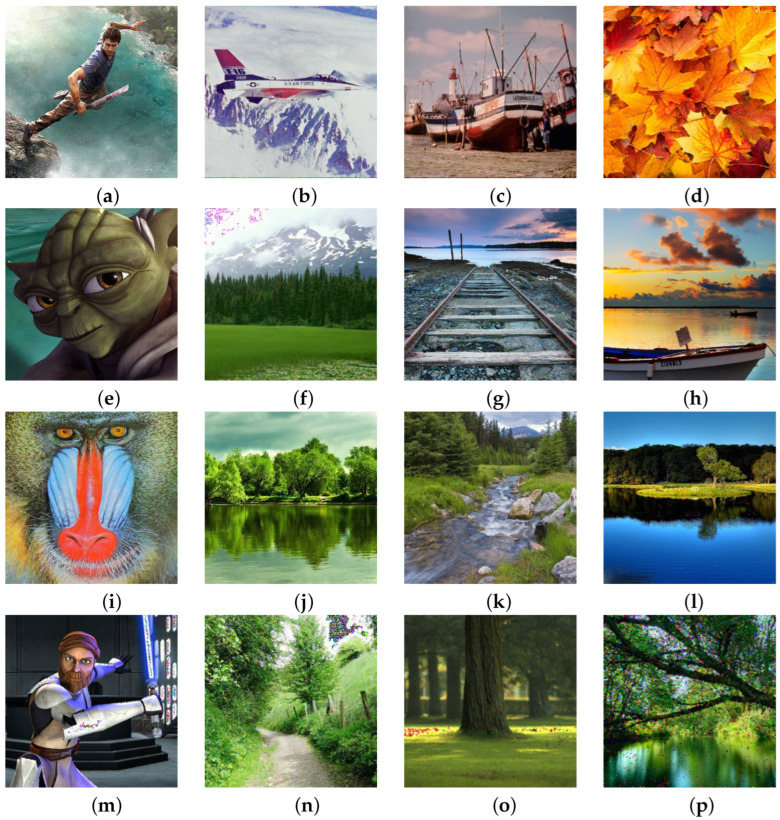
Results of regeneration filter treatment over test specimens from Figure 2.

The final presentation result is shown in Figure 11, where the processing by the Regeneration filter of all images with noise spatial densities as indicated in Figure 2 is presented. In addition to numerous numerical data, Figure 11 provides visual confirmation of the quality of the proposed filter across different noise spatial densities.

### 5.2. Discussion

In research conducted over the past five years, various approaches have been explored to reduce noise, with a particular focus on salt-and-pepper (S&P) noise. For instance, Quan et al. [19] developed an algorithm aimed at enhancing image resolution during rapid scanning through filtering, effectively removing S&P noise and increasing the spatial resolution of SICM scanning. This method combines a median filter with the Canny edge detection algorithm and edge interpolation to further sharpen the image. Zhou and colleagues [20] proposed a second-generation method for mixed noise removal in remote sensing, which combines extended convolution and an adaptive median filter to eliminate S&P noise while preserving image details. Lee and Jeong developed the Noise2Kernel algorithm [21], utilizing dilated convolution and a customized kernel for self-supervised learning without requiring clean/noisy image pairs, thus reducing dependency on masking during training. Feng et al. [22] explored vein feature extraction from finger images under S&P noise, employing principal component analysis and locality-preserving projections to improve recognition accuracy. Kwon et al. [23] developed a neural denoiser for PPG signals, leveraging deep generative models with transformers, while Fayaz and collaborators [24] integrated DWT and CNN for MRI image noise removal, employing a median filter to prepare high-density noisy images. Francis and team [25] applied a robust tensor methodology using simultaneous noise reduction through image decomposition, effectively removing high levels of S&P noise. Maleki et al. [26] used CDL and combined filtering to enhance agricultural surface data, allowing for improved segmentation and reduced S&P noise in CDL data. Li and colleagues [27] utilized SAR data for sugarcane mapping by applying time-series filtering to reduce speckle and S&P noise, thereby enhancing large-area pattern mapping. Liu et al. [28] applied LCAS-DetNet for ship detection in SAR images, using multi-local attention to reduce S&P noise and improve ship detection accuracy, while Wang and collaborators [29] employed ResNet-18 for concrete crack detection, reducing the impact of S&P noise with deep learning algorithms. Cao and Liu [30] proposed an enhanced median filter with entropy adaptation for high-intensity grayscale images affected by S&P noise, while Qin and team [31] used phase reconstruction combined with wavelet regularization for denoising complex noise types. Fajardo-Delgado [32] used genetic programming to remove impulsive noise from color images by applying pixel detection across color channels. Ilyin [33] utilized a hybrid Boltzmann model for nonlinear diffusion and S&P noise removal, and Zhang [34] applied a time-series and SNIC multi-scale segmentation-based method to reduce noise in crop structure extraction. Xue et al. [35] used Google Earth Engine for crop classification, reducing S&P noise and improving classification through radar image time-series. Wang and team [36] developed an iterative denoising method using mask symmetry to enhance the efficiency of S&P noise removal in grayscale images.

The proposed Regeneration filter offers several key differences compared to the approaches mentioned above for salt-and-pepper (S&P) noise reduction. Firstly, unlike most conventional methods that use median filtering as the basis for noise reduction, the Regeneration filter avoids using median or any other standard filtering technique. This provides a significant advantage because, while effective in removing noise, median filters tend to blur edges and reduce image sharpness, especially at higher noise levels. In contrast, the Regeneration filter is designed to process only noise-affected pixels, leaving untouched pixels unaltered. This approach preserves the original structure and details in the image, which is particularly important for applications requiring precise edge and texture retention, such as medical imaging and biometrics. A second important feature is the filter’s ability to selectively restore pixels based on local noise density, which enables high adaptability to different noise intensities. For instance, many of the described algorithms, such as methods using dilated convolutions (e.g., Noise2Kernel), genetic programming, or wavelet transforms, require predefined settings and specific models to optimize performance for particular noise types. These models often rely on pre-trained parameters and are difficult to generalize to noise that varies in spatial intensity. By contrast, the Regeneration filter employs adaptive pixel selection, where each pixel is evaluated based on its local environment. When high noise intensity is detected in a given area, the filter adjusts its processing approach to ensure more precise image reconstruction. A third major advantage is the modularity of the Regeneration filter, allowing it to be applied multiple times without degrading image quality. With most other methods, multiple applications of a filter can lead to further loss of detail or additional image blurring. However, the Regeneration filter is designed to support an iterative processing approach without additional quality loss, making it suitable for applications where high noise intensity is present. This is especially useful for very high noise levels, up to 97% spatial distribution. In such cases, most traditional methods have limitations regarding the maximum noise density they can effectively remove. The Regeneration filter has proven to be an effective solution even in extreme cases, thanks to its ability to adapt the processing range depending on noise density in the image. The fourth characteristic that sets the Regeneration filter apart from other methods is its focus on the noise’s immediate vicinity (near S&P, nS&P), enabling it to handle not only pixels with extreme values (0 or 255) but also those close to these limits. This approach further enhances reconstruction accuracy by enabling noise filtering in cases where extreme noise levels are present, while preserving subtle image details close to boundary values. Conventional methods, such as adaptive median filters, usually rely on fixed thresholds to determine noisy pixels, which can result in a loss of detail in surrounding areas, whereas the Regeneration filter uses customized algorithms that recognize fine noise nuances and adjust the processing accordingly. Another important distinction is the Regeneration filter’s flexibility and applicability across various contexts. Many approaches that use convolutional networks, dilated convolutions, or specialized processing methods are designed for narrow applications, such as crack detection in concrete or biometric signal processing. In contrast, the Regeneration filter offers a universal architecture that can be applied easily across diverse fields, including medical imaging, biometrics, satellite imaging, and other applications where preserving fine details is crucial. Its adaptive nature enables users to apply the filter across a wide range of images and noise densities without requiring specialized settings. At the end, the Regeneration filter is advantageous in terms of ease of application and processing, as it does not require model pre-training or complex configurations for each specific application. This makes it significantly easier to implement in real-time monitoring systems and resource-limited applications that require fast response times. Its ability to maintain image quality and details, even at high noise levels, positions it as one of the most effective solutions for S&P noise reduction across a broad spectrum of applications.

In addition to all the aforementioned methods, the results of the Regeneration Filter were compared based on key parameters PSNR and SSIM with works [37,38,39,40]. The results achieved by the Regeneration Filter demonstrated consistently high noise reduction quality and preservation of structural details in the image, with SSIM (Table 1) and PSNR (Table 2) values that are competitive with state-of-the-art methods based on neural networks and significantly better than traditional filters. Furthermore, in addition to standard digital image processing methods, the results were also compared with advanced techniques utilizing CNN models, further confirming the efficiency of the proposed algorithm.

The SSIM results (Table 1) clearly demonstrates the superiority of the Regeneration Filter in preserving structural information in images affected by salt-and-pepper noise. The algorithm achieves an average SSIM value of 0.9486, which ranks among the highest compared to other methods. It particularly excels at noise densities ranging from 10% to 50%, maintaining an SSIM above 0.98, which indicates a high level of image quality preservation. Even at high noise levels (90%), the Regeneration Filter sustains an SSIM value of 0.9041, outperforming most reference methods such as MDAMF (0.82) and SeConvNet (0.869). These results highlight the proposed algorithm’s ability to effectively balance noise reduction and preservation of critical structural elements in the image, making it well-suited for applications where high visual detail quality is essential.

The PSNR results (Table 2) further confirm the strength of the Regeneration Filter in effective noise reduction. The algorithm achieves an average PSNR of 32.465 dB, placing it among the top-performing methods. At low noise levels (10–30%), it attains PSNR values comparable to methods such as SeConvNet (45.1 dB at 10% noise), while maintaining consistent performance even at high noise levels. For instance, at 90% noise, the PSNR value is 24.7259 dB, outperforming methods such as NLSF-CNN (19.41 dB) and NLSF-MLP (18.93 dB). These results demonstrate that the Regeneration Filter effectively reduces noise with minimal information loss, providing a reliable balance between image quality and noise elimination capability.

The Regeneration Filter demonstrates superiority over traditional methods such as the median and contraharmonic filters by utilizing only noise-free pixels during the reconstruction process. In contrast, traditional methods involve all pixels within the submatrix, including those affected by noise, in calculating replacement values. This approach compromises the results, as noise-affected pixels introduce inaccurate information into the reconstruction process, particularly at high noise densities. The Regeneration Filter, leveraging multiple iterations and analysis of neighboring values, achieves more precise pixel reconstruction and preserves the structural characteristics of the image, which is essential for high-quality noise reduction.

## 6. Conclusions

In this study, we present a Regeneration filter, an effective method for reducing near Salt-and-Pepper (nS&P) noise in images that overcomes the limitations of conventional filters. The approach, based on localized analysis, enables selective noise treatment exclusively in affected pixels, without impacting unaffected parts of the image, thereby preserving edges and details essential for high-resolution applications. Our iterative processing method allows for multiple applications of the filter, where additional iterations do not degrade the image quality achieved after the first filtration, even at noise levels up to 97% spatial distribution. The filter’s performance was evaluated using standard image quality assessment metrics such as SNR, CSI, Entropy, PSNR, and SSIM to ensure that the results are measurable and comparable with other methods. Experimental results across various image databases confirm that our filter consistently maintains high image quality. The code was implemented in the R programming language, and both the data and code used in the research are available in a public repository, enabling full replication and verification of the results. Future research may focus on optimizing the filter for specific applications and extending the approach to other types of noise to further enhance the method’s applicability and efficiency.

## Figures and Tables

**Table 1:** Results of SSIM for different methods at different noise spatial densities (dataset: CBSD68). Tables present the comparative results of the Regeneration method with other approaches. The results for the method that achieved the best performance across the entire range of noise reduction are marked with *, while the results of the Regeneration method are presented in bold.

Methods	10%	20%	30%	40%	50%	60%	70%	80%	90%	95%	Mean
ACmF	0.984	0.973	0.96	0.946	0.928	0.905	0.875	0.832	0.76	0.692	0.885
ADKIF	0.984	0.973	0.96	0.944	0.924	0.897	0.859	0.809	0.741	0.685	0.878
ARmF	0.985	0.975	0.964	0.95	0.933	0.91	0.879	0.836	0.762	0.692	0.889
DAMRmF	0.97	0.959	0.948	0.935	0.92	0.9	0.875	0.838	0.773	0.705	0.882
DBA	0.96	0.95	0.94	0.92	0.88	0.84	0.77	0.67	0.51	0.38	0.782
IAWMF	0.987	0.978	0.967	0.953	0.936	0.915	0.887	0.846	0.777	0.713	0.896
NAHAT	0.99	0.981	0.97	0.956	0.94	0.919	0.891	0.852	0.789	0.735	0.902
NLSF-CNN	0.978	0.931	0.893	0.883	0.855	0.825	0.824	0.755	0.642	0.594	0.818
NLSF-MLP	0.944	0.898	0.861	0.831	0.804	0.777	0.803	0.736	0.626	0.579	0.786
MDAMF	0.99	0.99	0.98	0.97	0.96	0.94	0.92	0.88	0.82	0.71	0.916
SeConvNet	0.997	0.995	0.992	0.989	0.976	0.971	0.959	0.932	0.869	0.794	0.947
**Regener.**	0.9961 *	0.9952 *	0.9874 *	0.9813 *	0.9748 *	0.9642 *	0.9556 *	0.9271 *	0.9041 *	0.8 *	0.94858 *

**Table 2:** Results of PSNR for different methods at different noise spatial densities (dataset: CBSD68). Tables present the comparative results of the Regeneration method with other approaches. The results for the method that achieved the best performance across the entire range of noise reduction are marked with *, while the results of the Regeneration method are presented in bold.

Methods	10%	20%	30%	40%	50%	60%	70%	80%	90%	95%	Mean
ACmF	34.63	32.15	30.46	29.07	27.79	26.53	25.23	23.78	21.88	20.38	27.19
ADKIF	34.53	32.24	30.63	29.26	27.95	26.6	25.25	23.89	22.4	21.21	27.4
ARmF	35.11	32.62	30.87	29.42	28.07	26.74	25.37	23.85	21.9	20.38	27.43
DAMRmF	33.92	31.49	29.85	28.56	27.4	26.29	25.18	23.93	22.21	20.62	26.94
DBA	30.48	29.53	28.18	27.02	25.5	23.5	21.68	19.45	16.15	12.54	23.403
IAWMF	35.19	32.95	31.3	29.89	28.61	27.35	26.06	24.62	22.73	21.26	28
NAHAT	36.63	33.97	32.08	30.54	29.18	27.9	26.62	25.23	23.47	22.1	28.77
NLSF-CNN	34.32	30.87	28.5	27.37	25.86	24.49	24.21	22.29	19.41	18.38	25.57
NLSF-MLP	33.1	29.77	27.48	25.75	24.33	23.04	23.61	21.74	18.93	17.92	24.57
MDAMF	38.71	36.63	34.37	32.76	31.2	29.77	28.43	27.08	24.74	20.01	30.37
SeConvNet	45.1 *	42.64 *	39.7 *	38.19 *	34.87 *	33.82 *	32.2 *	29.82 *	26.64 *	24.38 *	34.73 *
**Regener.**	**41.8543**	**38.451**	**35.3442**	**33.9721**	**31.5934**	**30.127**	**29.0023**	**27.1144**	**24.7259**	**22.9**	**32.465**

## Data Availability

The code for the proposed solution, including all relevant scripts, implemented in the R programming language, is publicly available for review and replication. It can be accessed and downloaded from the following GitHub repository: https://github.com/RatkoIV/RegenerationFilter (accessed on 10 December 2024). This repository contains detailed documentation, example datasets, and instructions for running the code, ensuring that the findings reported in this paper can be fully replicated and verified. Additionally, the image database used in this study can be accessed at https://www.imageprocessingplace.com/root_files_V3/image_databases.htm (accessed on 10 December 2024), providing the original images required for thorough testing and validation of the proposed methods.
